# Metaphors or mechanism? Predictive coding and a (brief) history of empirical study of the arts

**DOI:** 10.1098/rstb.2022.0427

**Published:** 2024-01-29

**Authors:** Helmut Leder, Matthew Pelowski

**Affiliations:** ^1^ Faculty of Psychology, University of Vienna, Wien 1010, Austria; ^2^ Vienna Cognitive Science Research HUB, University of Vienna, Wien 1010, Austria

**Keywords:** empirical aesthetics, predictive processing, art, theoretical challenges

## Abstract

Predictive processing (PP) offers an intriguing approach to perception, cognition, but also to appreciation of the arts. It does this by positing both a theoretical basis—one might say a ‘metaphor’—for how we engage and respond, placing emphasis on mismatches rather than fluent overlap between schema and environment. Even more, it holds the promise for translating metaphor into neurobiological bases, suggesting a means for considering mechanisms—from basic perceptions to possibly even our complex, aesthetic experiences. However, while we share the excitement of this promise, the history of empirical or psychological aesthetics is also permeated by metaphors that have progressed our understanding but which also tend to elude translation into concrete, mechanistic operationalization—a challenge that can also be made to PP. We briefly consider this difficulty of convincing implementation of PP via a brief historical outline of some developments in the psychological study of aesthetics and art in order to show how these ideas have often anticipated PP but also how they have remained at the level of rather metaphorical and difficult-to-measure concepts. Although theoretical in scope, we hope that this commentary will spur researchers to reflect on PP with the aim of translating metaphorical explanations into well-defined mechanisms in future empirical study.

This article is part of the theme issue ‘Art, aesthetics and predictive processing: theoretical and empirical perspectives’.

*How has it come to be that we know rather more how people remember nonsense syllables and far less than we should like to know about how they create and understand art?* [1, p. 122]

## 1. Introduction

Predictive processing, or predictive coding (hereafter, PP), has provided an intriguing development in studies of perception, psychology and, as is illustrated in the present special issue, in art and aesthetics. PP is intriguing—currently more theoretically, although increasingly with empirical backing outside of visual aesthetics or art (e.g. [2–4])—for what it could promise. PP provides a unifying means of discussing the functioning or mechanisms, at their neurobiological bases, for how we might be actively responding to our environments and, as one aspect of this, to the arts. This is done, as is well-described throughout numerous papers both preceding and within this special issue (e.g. [5,6]; see also [7]), by considering mechanisms for the online processing, the storing and the anticipation (prediction) of certain schema or patterns—of basic visuospatial organizations, types or natures of responses, or, possibly, even higher-order cognitive structures, stored in the architecture of our brains—and the fitting of these to the world via sensory processes with the feedback generated from the matching process driving our subsequent sensations, cogitation and awareness.

Even more, when considering how this might be happening and from whence feedback would theoretically derive, PP suggests a theoretical reshaping of how we in fact conceptualize human processing experience [5,6]. PP does this by placing the emphasis not on successfully or fluently matching expectations, but rather on the value of deviations and error—not finding or perceiving what we expect—with these errors, met in a continuous, online fashion, being the means whereby we might actually perceive, update our schema or learn new things.

Although conceptually it is not a new idea that emphasis might be best put on ambiguity, difficulty or perceptual failure when discussing change, perception and growth (e.g. see, among many others, [8–14] for discussions in aesthetics; [15] for a good general psychological argument)—however, it is interesting that theorists seem to need to keep reminding us of this—PP also brought something even more intriguing to the table, making it especially impressive for the fields of cognitive and emotion neurosciences, and empirical aesthetics. This is achieved by making an argument for where and how this encoding, matching and updating might be accomplished in the brain. As now explored in a number of emerging papers—e.g. from retinal ganglion cells [16] modulating properties of the receptive field, to pyramidal cells connecting and potentially sending errors ‘up’ and predictions ‘down’ between sub-cortical and cortical regions [17], to areas tied to modulation of error detection precision (e.g. [18]) or even Dopaminergic system-related reinforcement learning [19]—researchers now have a growing, intriguing series of target areas and even mechanisms, offering a means to move from metaphor to actual empirical testing of the hypotheses and implications for how we meet and respond to our nuanced worlds, as suggested by PP. As put by Seth & Friston [20, p. 1], ‘predictive coding is a process theory with a biologically plausible back story and a considerable amount of (correlational) empirical support’.

In turn, studies have started to follow this promise. Early empirical studies with, say, salamanders [16] or looking into the Fusiform Face Area [21], have merged into studies on PP implications in higher-order perceptions, sensations, attention, motivation (e.g. [2–4,22]), music [23–26] and, again, even to visual aesthetics and art. To take only a few examples, Kesner [27] proposes to ‘leverage the [PP] framework to elucidate the complex issue of why and how people fail to meaningfully connect with modern and contemporary artworks’ (p. 1), or Vuust *et al*. [28] claim, even more universally, using music perception as their topic, ‘we show that music perception, action, emotion and learning all rest on the human brain's fundamental capacity for prediction—as formulated by the predictive coding of music model’ (p. 1). This also fits with a large body of previous work that suggests the promise that PP is the mechanism for uncovering a range of aspects that often draw us to art—ambiguity and negativity [29], emotions [30]—and general arguments for our appreciation of art itself [31,32].

We are happy that PP has opened exciting arguments in aesthetics, with suggestions that this might unlock how we perceive and respond to art. At the same time, however, while the promise of PP is slowly advancing from ‘biologically plausible’ to concrete, the movement from metaphor to actual mechanism appears even slower, especially in aesthetics research. This is most probably due to a number of reasons—from basic lack of critical researcher mass in empirical or neuroaesthetics when compared against other domains of perception or neuroscience, to the differentiated and plain different modalities of types of art.

For example, in comparison to music, Kesner [27] suggests that ‘works of visual art do not possess anticipatory structures and statistical regularities comparable to linguistic or musical syntax, which seem to provide a better fit with the hierarchical structure of predictive error minimization’—a claim with which, at first consideration, we would tend to disagree (see e.g. [14,33]), while also agreeing that such questions still need to be asked and addressed when bridging with PP.

Perhaps even more, as also suggested by this preceding comment, is the realization of the complexity, the nuance and the ambiguity involved in our aesthetic engagements and meetings with especially visual arts [33] making it hard to test sequential predictions, errors and corrections hypotheses (see [7]). Indeed, a look at the history of empirical or psychological aesthetics is saturated with insights into this challenge, but also with the great promise of why we should care about the arts. Interest in art and aesthetic experiences was already present when empirical psychology was founded as a field of research. In response, the field is also characterized by a set of theoretical arguments that provide metaphorical guidelines for how we might approach this task. Some also anticipated ideas such as PP. However, they also tend to elude translation into a concrete, mechanistic operationalization, once again similar to today's discussions on art and PP.

Thus, in this short theoretical commentary text, we aim to bring awareness to this difficulty in creating a convincing implementation of PP in aesthetics or arts, as long as the field of empirical aesthetics is permeated by metaphors—e.g. as we will see below, aesthetics ‘from below’, ‘ascending stages’, good gestalt; human-art blending to create atmospheric content—that evade strict operationalization regarding how cognitive, perceptually, biologically, these might actually come about. We do this via a brief historical outline and discussion of some main historical developments regarding this area of tension. Although not yet providing concrete empirical solutions, by looking at these chosen examples of psycho-physiological aesthetics and attempts to explain art appreciation psychologically, we argue that we might provide one more lens for considering how PP is not completely new but is still in need—and search—of paradigms and operationalizations for proper testing its mechanisms and their biological underpinnings. Throughout this paper, we focus on the visual arts or visual aesthetic discourse, as this is the area most overlapping with these historical examples and has the most outstanding need for consideration regarding the metaphor-to-mechanisms topic (although we of course refer the reader to many important papers in other domains such as, e.g. music; [24]; see also other contributions to this special issue). We end in some brief suggestions for future research.

## 2. A (brief) historical search for elements of predictive processing in the domain of art and aesthetics

Since the beginning of empirical psychology, researchers have recognized the intriguing nature, but also the challenge, of aesthetic experiences and art, as a psychological experience.^1^ Gustav T. Fechner, the founder of empirical psychology, as well as—because he used this as one of his original points of emphasis—empirical aesthetics, examined psychological correlates of preference and (aesthetic) sensations specifically, using very simple materials, such as rectangles to measure preferences for the Golden Section proportion. On the other hand, in his ‘Preschool of Aesthetics' [35], he already recognized and named the different levels of the effect of works of art. As he noted, ‘In works of art, where an entire structure of higher relationships over lower ones, with a conclusion in the idea of the work of art, the variety increases with the height of this structure, not only by virtue of an increase in the differences in the underlying sensuous material, but also in the stages of the relationships ascending above it, in short, not just in terms of width but also in terms of height’ (p. 70, translated by HL). Of course, in the use of terms such as ‘ascending relationships’, ‘height’ and ‘width’, one could argue that he also provides us with a number of metaphors without really moving to specific psychological (much less neurobiological) operationalization.

The interest in the nature of aesthetic experiences of art, and the complex blending of processes, of course long preceded this research founding, largely as a main topic of philosophy and other related discourse (e.g. [36]). However, with the emergence of aesthetics as an empirical science, the need for at least thinking of operationalizations in terms of defining what can be measured shifted the discussion away from heuristics to empirically bounded definitions, trading a lack of abstractness for an empirically testable substantiation. See, for example, the term ‘aesthetic value’, as discussed from its historical roots by Shusterman [37], which in order to be studied in empirical sciences, had to be transferred into a behaviour, be that a key press to indicate a value on a scale or an amount of money someone fictionally would be willing to pay [38]. In turn, in his combination of aesthetics ‘from below’ as a bottom-up approach to art and aesthetics, from sensory stimulation, to perception and the explicit top-down influences of ‘the ascending stages’ Fechner, too, already set the ground for interactive understanding of the complex aesthetic experiences. As we still see in our current approaches to explaining art appreciation, this posed a metaphor for conceiving interaction as a bottom-up processing of sensory perception, where mismatch and error require top-down activities (e.g. [27]).

This above reasoning can, of course, be said to present one of many precursors to PP. At the same time, Fechner also made manifest—through his interest and arguments for why art/aesthetics were intriguing, but also through what he actually investigated and what this could possibly hope to reveal about the underlying processes—for the tension between metaphor and mechanism in art-focused discourse. Even during Fechner's time, it was already recognized that the sheer breadth of decisions and factors that go into art-making, as well as its open-ended nature, make it particularly difficult to study empirically [39]. And thus, he resigned to studying basic responses to figures or patterns, with the idea that someday the actual top-down and bottom-up blending mechanisms might be achieved.

From this beginning, the epistemological status of visual art, as discussed by Fechner [35], also further introduced complexity, regarding an awareness that the requirements of perception of visual aesthetic/art examples did not automatically aim at a veridical, verifiable representation of reality as in many other cases [40]. The peculiarity of artistic representation towards the beginning of the twentieth century—with artists exploring approaches moving from the mimetic to the more and more visually and conceptually abstract, and thus increasingly detached from concreteness and objectivity when considering reception—also inspired empirical aesthetics in terms of free design, fantasy, free narration and deviation from reality. As such, at another main point of transition, Wundt [41, volume 3], in his ‘folk psychology’, examined the various components of artistic production with regards to posited psychological mechanisms and function and embedded as the core a kind of cultural psychology for understanding human action and development itself.

As an example of the above line of argument, Wundt considered how psychological features might explain the emergence of landscape paintings as a representation of the inner state of a person. ‘Now the landscape is no longer the atmospheric background to a picture in which the human being is the centre, but the human becomes the scenery's accessories in order to increase its atmospheric content. As not reality, but the effect on imagination and feeling is the norm she follows here’ (in [41] ‘Die Kunst’, p. 303).^2^ This is but one of the examples in which Wundt explains the actual effect of art through reliance on emotion triggering elicited through artistic features. These are discussed on various levels, from sensory stimulations to the formation and perception of shape, style, artistic change and very distinctive inventions in terms of phantasy. However, Wundt also did not, in many ways, move on to providing explanatory mechanisms for these above effects or even incorporation of features of art. His explanations seem highly metaphorical, describing experiential phenomena, not psychological processes, let alone any biological implementations. The contrast to the multitude of psychophysical studies carried out in his laboratory, which described psychophysical laws with great precision, leaves the reader somewhat astonished.

Of course, there are many arguments for the lack of movement from metaphor to mechanism in Wundt's discourse. From Wundt's perspective, psychology was concerned with conscious experience or the topic of the conscious mind, not psychological processes as would be understood today. Wundt further believed that the basic building blocks of such experience—sensations and feelings—could be studied in the laboratory with basic methods. Whereas, for higher mental processes, such as thinking, or art and aesthetics, he argued that experimentation and the laboratory were wholly inadequate. These aspects, Wundt further argued, were so dependent on meaning, culture or life experiences, that a different approach was required.^3^ Relatedly, Martindale [1] offered an interesting interpretation, suggesting that in the *Völkerpsychologie*, Wundt tried to demonstrate the discipline of psychology as the main unifying foundation of many sciences, with aesthetics and art being only a good example to demonstrate that psychology is necessary (although the ‘how’ appeared to still be forthcoming) to understand all cultural activities.

Such an ‘imperial’ possibility of psychology was not successful nor sustainable [1]. Apparently, however, Wundt was looking for a unified view of art and culture that had its origins in the basic psychological processes that can be described by psychophysics. PP also faces similar challenges, precisely at the point where it describes the transition to the higher cognitive processes and the danger of the definition of the postulated processes being postulated must be avoided at all costs. Looking back today, one main argument for Wundt's difficulty, which might be applied to the present discussion, was perhaps because the collection of examples and considerations he presents lacks a uniform traceability to basic mechanisms that psychophysics makes so strong. The psychophysics that Wundt pursued still provides the conceptual framework for experimentally testing the adjustment screws of PP in the perception process. However, this was hardly ever made explicit in relation to his cultural theories. Nevertheless, also in Wundt's meeting of metaphorical aims and reality of testing, there is already a hint of a path in which actual psychology, which, for example, turns to a variety of individual effects, may increasingly lose sight of the possibility of a unified theory. Wundt did not succeed in this either, which is why he hardly plays a role in psychological theory (including empirical aesthetics); in the history of empirical aesthetics, gestalt psychology was more likely to achieve this.

The first half of the twentieth century, in some ways, was a rather fruitless time for empirical aesthetics, mostly due to the prevalent emphasis on behaviourism, with the obsession of restricting science to only observable behaviour or outward acts, which naturally saw very little research interest in phenomena that were more demanding of complex perceptual processes or muddled by emotion or other affective/body response, and thus, as Martindale [1] summarized, ‘best seen as a disaster for the discipline of psychology and an unmitigated disaster for psychological aesthetics' (p. 123). This is, of course, a massive exaggeration that ignores many advances in, among other areas, empirical aesthetics of music, assessments of affective value in terms of tones, intervals, cadence, as well as an emergence of interest in basic visual form-related preference, impacts of context and testing of related mental processes (see [42]).

One theory that undoubtedly had the strongest connections to PP at roughly this time was gestalt psychology, which flourished at the beginning of the twentieth century. Gestalt psychology often gained its persuasive power by phenomenologically showing how deviations between physical and psychological reality (i.e. errors in perception, visual illusions) reveal the underlying mechanisms of our perceptual processes. This referred back to unconscious interference postulated by Helmholtz [13] by which conclusions must be drawn in the perception system and thus, so to speak, components of the mind, but represent reality outside of the mind itself. For aesthetics and art appreciation, gestalt approaches mainly became salient through Arnheim's [43] work, which for decades set a relevant contribution.

As Arnheim [43,44] stated ‘perhaps perception consists in the application, to the stimulus material, of “perceptual categories”, such as roundness, redness, smallness, symmetry, verticality, etc. which are evoked by the structure of the given configuration’ [44, p. 32]. Thus, he provided a translation of Fechner's processing from below, while further arguing ‘if perceiving consists in the creation of patterns of perceptual categories, adequate to the stimulus configuration, and if the artist's task includes the representation of such patterns, then he has actually to invent a pictorial form that more often than not cannot simply be “read off” from the percept’ [44, p. 35]. Rather, higher processes are needed to accomplish an aesthetic experience, and one may have to go through some struggles (or error/mismatch) to accomplish an appreciation. This contribution of gestalt psychology can be seen particularly in the large body of visual illusions it helped to discover and/or explore, or moments in which sensation and perception obviously do not correspond to physically measurable expected entities. For example, in the classic ambiguous double images, such as duck–rabbit or young–old women, the sudden change in what is perceived can be seen and explained by binocular rivalry, which is also often found in art. This makes gestalt psychology an interesting historical approach, which not only described but attempted to explain holistic, but complex phenomena encompassing perceptual experiences, especially through the focus on misguided, obviously error-prone perceptions.

With PP, of course, there is also the assumption and frequent verification of the situational, or repeated, adaptation to changing conditions, which are interpreted in the sense of flexible and adapted prediction systems. Perhaps even more intriguing, looking to the original basis for the interest in its sets of optical illusions discussed in gestalt ideas, these are often accompanied by moments of surprise as an affect, the ‘aha’ experiences or simply, sudden insights (see [45]). And indeed, these are also described in the current work on PP as switching points of a possible aesthetic experience through PP. As put by Van de Cruys *et al*. [7], for example, ‘our hypothesis is that the specific dynamics of uncertainty resolution and our expectations thereof will determine the intensity of the emotion’ (p. 10). This is also in accordance with Arnheim [44, p. 45] when he assumed that ‘the organism does not tend simply to equilibrium. It strives to obtain a maximum of potential energy and to apply the best possible equilibrium to it. Aesthetically, this may correspond to the odd formula of unity in variety.’ Of course, once again, we should be reminded here that gestalt, as in the arguments above, has struggled to move from metaphor and very nuanced, well-described models to actual verification of more than basic neurobiological mechanisms.

Indirect empirical evidence that emotional response is stronger when challenging art is processed has also been found in emotion measures via facial electromyography by, for example, Leder *et al*. [46], who showed that, compared to non-experts, experts tend to show less extreme valence ratings and corrugator supercilii activations and also ‘like’ negative art more. Such a finding would be in accordance with arguments that, in this case, expertise may have changed the expectations/schema structure and, when applied, in consequence the emotional effect of disturbing, negative art. Moreover, regarding the emotional processing of ambiguity, a state not much appreciated in everyday life, Jakesch *et al*. [47] found that zygomaticus major muscle activation was higher and corrugator supercilii activation was lower for ambiguous versus non-ambiguous versions and suggested that this might ‘reflect a positive continuous affective evaluation to visual ambiguity in paintings’ and further suggested this as ‘indirect evidence for the hypothesis that visual stimuli classified as art, evoke a safe state for indulging into experiencing ambiguity, challenging the notion that processing fluency is generally related to positive affect’ (p. 1; see also [48,49] for similar findings relating ambiguity or challenging art to the brain). As one example from the emerging PP discourse, in their paper, Van de Cruys *et al*. [7] present a simple ambiguous Hebb figure to illustrate a cycle of processing stages as ‘processing errors’ that meander between ambiguity (i.e. prediction errors) and resolutions at different levels. While their description of processes alternating between insight and questioning phenomenologically is sound, the necessary processing stages described are again, and much as in gestalt psychology, not yet empirically tested.

One idea for where we might go or learn from here, is contained in the idea that the descriptions above contain some mental construction elements, in the sense that processes such as sudden interpretation of a line belonging to a shape (e.g. of a profile) could be quite complex processes as described in the binding-model of Hummel & Biederman [50], but might also vary from person to person, as a result of experience-based, learned top-down priors. The challenges for empirical aesthetics are the experiments that allow us to measure these processes. Gestalt psychology offered numerous phenomenological examples that might inspire future paradigms, protocols and tasks.

In Empirical Aesthetics since the 1970s, the focus has moved to identifying variables that make artworks or patterns appear beautiful, preferred, desirable or emotionally moving. Berlyne, in his ‘New Experimental Aesthetics' [51], described his arousal-based approach as ‘the appeal of a work of art depends on the interplay of two sets of factors, one tending to drive arousal upwards and the other tending to reduce arousal or to keep it within bounds. The idea that aesthetic value requires a combination of two partly opposite and partly complimentary factors has cropped up repeatedly over the centuries’ (p. 9). He aimed at a unifying approach explaining aesthetic appeal through arousing potential, which in turn he argued was determined by three types of characteristics—collative, psychophysical and ecological.

How do these classifications refer to the current topic of PP for art? Inspired by Berlyne, the role of variables such as finding meaning has pushed empirical aesthetics towards a cognitive, higher-order cognitive level of processing, in the sense that most current theories refer to these kinds of explanations. In this way, though strictly psycho-physiological in its origin, Berlyne has shown the way to trace different levels of processing, more bottom-up and more top-down, to a uniform underlying construct of the stimulating arousal potential. If this emotional component is seen as part of PP, then at least his structuring according to variables is inspiring for a unifying theory of PP to explain art/aesthetic processing.^4^

Empirical studies in this context today might be revisited from a PP perspective: for example, regarding visual principles in art, Locher & Nodine [53] studied the role of symmetry, measured visual exploration of original and altered artworks with eye-tracking devices, and found that ‘the presence of dynamic symmetry in an artwork enhanced visual exploration of it’ (p. 482). They interpret this finding with Berlyne's explanations of visual exploration. However, the result could also illustrate the need to resolve more complex, and more uncertain, predictions in a composition that cannot be grasped in a holistic, single-glance fashion. As Clark [5, p. 190] states ‘attention fits very neatly into this emerging unified picture, as a means of variably balancing the potent interactions between top-down and bottom-up influences by factoring in their precision (degree of uncertainty)’. Although, again, Berlyne serves as a perfect example of the metaphor-meets-mechanisms impasse. As he concluded in his seminal work, and as could be used as a similar argument against PP-related studies, ‘sooner or later’ the ‘“synthetic” [one might say metaphorical] approach must … be supplemented with “analytic” studies, in which reaction to genuine works of art are investigated with a view to unravelling their determinants. This, however, involves difficulties. Any two paintings, for example, must differ in at least a thousand respects. If we find a reliable difference between reactions to the two paintings, any one of these factors, or any combination of them, could be responsible for the difference’ [51, p. 182].

Since the beginning of the twenty-first century, various theories of empirical aesthetics have similarly aimed to explain processes involved in aesthetic experiences. Often in the form of simple box models, these offer a more systematic way to consider PP-related aspects. The underlying attempt to describe the complex momentary experience, the enjoyment of a work of art, the joy of a beautiful view, in terms of processing stages of perception and higher cognitive components, of course, if PP is valid, must be understood in terms of their main underlying principles. As reviewed recently (see e.g. [54, p. 2]), most ‘psychological models of aesthetics, and more specifically, art processing generally have three main components … : (i) inputs that feed into experience… the personality of the viewer, social or cultural setting, background affective state, another context … as well as the specific artwork body and its history; (ii) processing mechanisms, which act on the inputs in specific stages (explained further below) and (iii) mental and behavioural consequences (outputs) that arise from processing art’. Further, ‘in general, these models and theories tend to imply a directionality from initial bottom-up information to more cognitive top-down processing. More basically, these imply that we somehow “take in” or “receive” information in these stages, which we then “do something with”, “decode”, or fit to schema/past experience’.

This taking in, matching and responding of course at least suggests an interest in—if not an actual discussion of how this would be accomplished (see e.g. [14])—many processes similar to those implied in PP. Specifically, models often contained some aspect of pre-expectations or even schema, with the realization that these must be a guide for subsequent processing. Interestingly, the one-way nature and the taking-in aspect can, and has, also been noted as one of the reasons why PP might have been such a revelation once again, suggesting against fluency, success and matching-related standpoints (see e.g. [14]).

At the same time, models also often did note that the degree of a match (often discussed in terms of fluency, meaning or understanding) would certainly lead to differing results. The discussion of model processing results also calls forth many aspects that might be discussed in PP, such as: ‘capability to influence basic aspects of affect or the body’ (see [54, p. 2] for review), and possibly stemming from a wide range of aspects such as specific emotions/moods evoked by content or derived from the act of viewing; physiology, such as heart rate, skin conductivity or other processes of the autonomic nervous system; actions, gesture, eye movement or physical movement during art reception; appraisals or particular judgements (beauty, liking); meaning-making, novelty and/or ‘art['s ability to] impact what we see, induce changes in visual or perceptual experience involving new attention to physical aspects’.

A model that provides a key example, encompassing much of the earlier work and also a still go-to basis for describing especially these initial and visual or sensual processing and meaning-making aspects of experience, is by Leder *et al*. [33]. This essentially describes a five-step sequence of cognitive-affective processes. The first stage describes that a work of art, a painting, is first perceived through the senses. Actually, as discussed throughout the papers of this special issue, here a special status might be stated, as often, e.g. in the museum, the next artwork on the wall offers deliberately new, therefore hardly predicted, perceptual experiences, and as a consequence, art is often visually, sensorially interesting; moreover, the perceived errors are not harmful, but rather stimulate pleasurable, aesthetic processes to reduce the prediction errors. The early stage provides the material—the, so to speak, ‘visual stimulation’—which builds up the various levels of aesthetic experience of art.

In terms of time, a first modulation of expectations and the fulfilment of expectations was argued to actually take place before looking at the work of art, which means that the viewer can adopt a different basic attitude. Cupchik *et al*. [55] have distinguished in this context the everyday perception, which aims at object recognition, from the aesthetic, ‘in contrast, when viewing visual images as artworks, we also tend to experience subjective reactions to their stylistic and structural properties’ (p. 84). In the Leder *et al*. [33] model, the challenge of art perception is presented as analogous to a kind of problem-solving. Ideally, the goal of perceiving is to go through a successful, satisfying process of understanding that ultimately leads to a satisfactory finding of meaning.

In the original model, this mastery was formulated in a quite normative way, as if there was something right, correct, to discover. Of course, it is now clear that this process is essentially characterized by much more nuanced sets of factors, the resolution of which is often not successful in art or remains subjective in a certain way (i.e. cannot be formulated discursively as in a justifiably correct solution). The character of ambiguity has already been mentioned, but its importance was probably underestimated when the model was originally formulated. As put by Leder *et al*. [33, p. 499], ‘the processing stages Cognitive Mastering and Evaluation are closely linked as these two build a feedback-loop. The results of the cognitive mastering stage are permanently evaluated in relation to their success in either revealing a satisfying understanding, successful cognitive mastering or—expected changes in the level of ambiguity’.

The above evaluation stage may, therefore, guide the aesthetic processing by measuring its outcome in terms of PP, finding a new model of prediction. Knowledge about the context, and learned models of prediction, all make these processes special, if not idiosyncratic. Art expertise, making use of different layers of processing, such as using style and visual features of the artwork, add levels specific to the art experience, different from the way that art-naive viewers process artworks, and who often refer to content or external referents (e.g. [56,57]). In addition, there are clear indications in these cognitive-affective explanations of aesthetic experiences that the individual's learning history is an essential factor in the art experience. This realization in research in empirical aesthetics can be seen in the increasing number of studies on the effects of expertise. However, as is often the case, the operationalization of the components in many of the models above also present main challenges for our empirical studies, and researchers argue against the very efficacy of box-arrow models at all (see e.g. [58]). Again a challenge and a potential for, perhaps, PP.

In parallel to the Leder *et al*. [33] model, an early extension of stages of information processing into the domain of neurosciences was provided by Chatterjee [59], and in the last two decades, numerous new findings regarding art-related brain networks have been published; how these correspond to the unifying mechanisms postulated by PP also seems promising. For example, the finding that the Default Network [60,61] plays an important role in aesthetic experiences refers to a state in which change is prepared by the suppression of certain action tendencies that might be formed by the context of experience, and thus explain the special status of art in human culture. In this respect, the special brain state at least implied by such findings might be seen as the neurophysiological condition to what Van de Cruys *et al*. [7] propose as ‘the friction of prediction error creates the potential to (unexpectedly) make progress in structuring the stimulus, and, when this structuring happens by means of one's own epistemic (cognitive-behavioural) faculties, it sparks positive affect and situates the discovered structure in the outside world’ (p. 14).

Tinio [62] also proposed an approach to psychological understanding of art through the interplay of two reversed sequences. ‘According to the model, aesthetic experiences *mirror* the art-making process in the sense that the early stages of aesthetic processing correspond to the final stages of art-making’ [62, p. 265]. These processes are usually reversed in art reception (e.g. [33]). In this respect, the late stages of aesthetic processing correspond to the initial stages of art-making. Tinio optimistically summarizes that by ‘considering the aesthetic processing of an artwork in terms of the artistic processes that produced it allows for an account of the experience of art in its fullest manifestation: one that could be self-referential, pleasurable, challenging or even repulsive’ (p. 265). However, again, the operationalization of components remains a target for future empirical studies, yet the sequence of stages systematically described makes this model an ideal starting point for our understanding of where in the empirical aesthetic process fit and no fit, possible deviation and prediction errors might be expected.

Finally, one last model that perhaps is relevant for providing a link to PP, is the Vienna Integrated Model of Art Perception (VIMAP), Pelowski *et al*. [63] (see also [14]), which provides an attempt to describe underlying pattern of schemata, their challenges and how their adaptation not only fosters learning, but even transformative states. This was created as a combination and extension of previous models [33], focusing on the integration of both bottom-up, artwork-derived aspects of the visual art image as well as, importantly, secondary, viewer-centred, processes in which a viewer may respond to, give meaning to, or even modulate their initial responses. This also posited several processing stages, beginning with those presented by Leder *et al*. [33], leading to cognitive mastery. However, rather than a simple positive/negative output, here the model introduces new elements that also coalesce with the PP topic. This involves, first, a more detailed discussion of the prior expectations or classification individuals bring into an engagement. While many models of aesthetic processing had acknowledged that individuals must certainly have expectations and that these should play a key role in experience, the VIMAP argued that by articulating these more explicitly would give a basis for better understanding how they are applied and changed within experience.

We argue ‘Before a perceptual activity, viewers already hold a set of postulates directing behaviour, perception, expectations for interaction, and a viewer's likely response to the outcomes of action’ [14, p. 84]. In turn, the model employed a hierarchical structure (following an earlier cognitive discussion of Carver [15]), with a collection of ideal traits and concepts that the individual aspires to and that are integral to their identity at the head, branching into more general schema for pursuing these traits, and which are divided into more and more specific ‘action goals’ for behaviours and perceptions—with the relative position (top to bottom) or interconnection of postulates signifying increasing/decreasing importance or threat to the self. By connecting the core expectations at the head of the model to low-level schema, this structure provides a frame whereby all actions or perceptions entail the application of this structure to the reality or scrutiny of the environment. By considering the repercussions of this application through protection or modification of the self, this gives a shared basis for considering the following processing outputs.

Interestingly, this arrangement of schema—although metaphorical in the model—relate nicely to PP. As put by Clark [5, p. 181], for example, ‘the brain stores schema (“hypotheses” and “beliefs” about the world)’. These are not ‘consciously held mental states’, but neuronally encoded probability distributions about what kind of sensory information might be found. This argument continues: we use (from the beginning) top-down knowledge to generate a kind of ‘virtual version’ of the nature of the world, which we apply, actively, as a component of perception or other processes in order to act, perceive and understand. This fits what is argued for in the VIMAP, which suggests that it probably is the aim of individuals in most experiences to find a good match to the schema they have—either by truly matching expectations or by not looking terribly hard (i.e. employing only low levels of schema structures) as this indeed might allow for smoother processing of experience.

At the same time, much as with PP, it is in fact when there is a mismatch that interesting results might be found. In turn, the model posits a specific component that can help to account for this in the initial mastery stage of experience. This involves a ‘congruency check’, where an individual would consider the relative match of the task or processed information with prior schema, and involving a number of information levels, where the individual moves between multiple interpretations. We also included, and which also can be found in PP, a check for ‘self-relevance’ or of the broader implications of this processing to their own schema structure. This would also identify—or even help to determine—the relative sense of distance (i.e. a personally detached ‘aesthetic’ versus ‘pragmatic’ mode) that could be employed. A parallel can be found to PP in the amount of specificity we employ in error detection. Stefanics *et al*. [29] give a metaphor here of setting the gain on a radio receiver—adjusting, depending on context: whether we assess a coin in our pocket based on colour, if looking to pay a fee, or requiring us to carefully read its details, if looking to add a rare example to a collection—determining how close we look for difference.

These checks then lead to five suggested outcomes, which might also be targets for PP: (i) high congruency and low tie to the self, and a default or even ‘facile’ outcome with art; (ii) an outcome involving a sense of ‘novelty’, insight, or heightened interest, tied to low self-relevance but lower congruency because of discrepancy or difficulty in processing experience; (iii) high schema congruency in conjunction with higher self-relevance leading to experience in which some element—artwork form, meaning, the situation, even the experience of perceiving—resonates with a viewer; (iv) ‘negative’ outcome with viewers feeling an implicit awareness of self-threat, attempting to extricate themselves from the situation via covertly changing the conditions of their task or environment so that the discrepancy can be better assimilated or ignored; (v) self-relevance, low congruency, where rather than escape, individuals create a new means of addressing the discrepancy or object itself, leading to transformative experiences.

Thus, as with many of the discussions above, this model also both overlaps with PP but also raises new arguments. That is, the argument for novelty, challenge, transformation, even the facile outcomes, in conjunction with the specific (albeit metaphorical) mechanism posited, give one more argument that does seem to anticipate or make the same arguments as PP. It also raises questions about when and if schema match might be desired—in cases of harmony, resonance, absorption, flow. Even more, it suggests new areas for PP to explore—describing not only how we might enjoy fluency or even the existence of art, but also profound powerful states. Van de Cruys *et al.* [7] beautifully describe such an effect. ‘Art does this not by painting a rosy picture of reality, nor by simply yielding to our prior expectations about the kind of world we should encounter. Tension, dissonance and expectation violations are part and parcel of good art; as has been noted since the earliest philosophical writings in aesthetics. Indeed, the genuine moments of discovery that we find in art demand tensions and uncertainties (change) as much as they demand closure and certainty (order). The order reached through art is moreover often a stimulus for further forays into change: it acts as a self-validation that gives us the freedom to venture into a capricious and precarious world again, exploring new environments and ways of being (*change*). … This is why aesthetic experiences are often also considered transformative, ethical experiences: they shape (freedom of) action.’ Here as well, we also find some emerging empirical evidence, building (if slowly) from these metaphors or theoretical framework to empirical findings, which also call to mind key aspects of PP. Studies have found (at least self-report) evidence for movements through posited model stages to generally profound, emotional or transformative outputs (e.g. [64,65]), and which also foregrounds aspects such as schema mismatch and reflection. See also new findings from Vessel *et al.* [66] showing the importance of self-relevance in appeal with art and which might be more directly related to PP. Once again, however, the real challenge for this, and all of these approaches, lies in making this connection from useful model/theory to mechanism itself.

## 3. A few notes on next steps for predictive processing and empirical aesthetic research

To conclude, this compilation of theories from empirical aesthetics was intended to show which paths have already been trodden, but also which challenges still have to be mastered in future research. In this vein, we might end in a few suggestions. First, it is useful, even as we are intrigued by the ever-present newness of models and theories—even those that promise new insights for how theory and neurobiology might relate—to also remain cognizant of the challenge and the demand that we continuously seek of empirical evidence. Metaphors, while valuable in conveying abstract concepts and providing epistemic value, do not in themselves constitute scientific explanations of the precise patterns of neural activity underlying an aesthetic (or any) experience. Researchers should be mindful of multiple inherent questions—How can promising theory actually be tested at the level of body, perceptions; the brain? How do we connect models to empirical evidence? How can we keep ourselves from stopping at the level of metaphor in this search—what are the limitations if we do not? At the same time, as shown in the unique challenge of our engagements with visual arts and aesthetics, just because finding elegant empirical solutions or explanations is difficult, this should not mean that we abandon such as challenge. The very intrigue of the arts, and its ever-presence through psychological history, shows the huge importance or knowledge that might come through its investigation. Here, attempts to model more profound, awe-inspiring, transformative experiences might provide key future targets, as well as the involvement of ambiguity or self-relevance, for truly next steps in PP assessments. Such investigations may also help to illuminate other challenges. For example, while PP offers a promising theoretical framework, the difficulty in translation into concrete, mechanistic operationalization and the somewhat simplistic aspects that are currently good candidates for research could limit its applicability in practical contexts. Similarly, questions should be asked regarding generalizability across different artistic forms, cultural contexts or individual differences in aesthetic preferences.

Much as PP might provide new insights into the arts and aesthetic experience, due to their complexity, the arts might also shed new light on PP processes. Understanding aesthetics involves delving into the intricate realm of cerebral activity, which is intricately coordinated within a living, embodied agent situated in a dynamic environment. The arts might also pose other angles on PP discussions—such as the role of the body or the relation between interoception and aesthetics in an embodied approach (see e.g. [67]). Interestingly, there is also a recent study [68] which looked at engagements with installation art and reported correlations between aspects of mismatch regarding ‘proprioception’ (awareness of body, movement, balance, etc.) and especially ‘disturbance’ (feeling awkward, unable to move, etc.) as a correlate to transformative experience, and which again calls to mind PP. As is highlighted throughout this issue, the translation of PP into concrete, mechanistic operationalization poses a significant, yet rewarding future task. In this sense, although empirical aesthetics is still permeated by multiple metaphorical explanations, the challenge lies in where we go from here.

## Data Availability

This article has no additional data.

## Endnotes

1 We are aware of the complex interaction between discussions of ‘aesthetic’, as a general mode of perception, cognitive stance or affective evaluation, and ‘art’ or ‘the arts’ as a field(s) often named as prototypical for aesthetic discourse. We use both as possible topics for PP here, rather interchangeably, as do many authors we reference, without going into the long discussion of ontology and semantics (but see [34]).

2 Original quote: ‘Jetzt ist nicht mehr die Landschaft der stimmungsvolle Hintergrund zu einem Bilde, dessen Mittelpunkt der Mensch ist, sondern dieser wird zur Staffage der Landschaft, um deren Stimmungsgehalt zu steigern. Denn nicht die Wirklichkeit, sondern die Wirkung auf Phantasie und Gefühl ist die Norm, der sie hier folgt.’ Translated by HL.

3 We would like to thank Marcos Nadal in the peer review of this paper for pointing out the above arguments.

4 We note of course that, beyond discourse on aesthetics, many other salient metaphors have greatly impacted psychology, neuroscience and art/aesthetic discourse, such as the test specified by Turing and/or Fodor's [52] ideas regarding modularity of the mind, the impact of parallel distributed processing on connectionism, as well as a constant recurring pattern of adopting the cutting-edge technology of the day as a toolkit for comprehending cognitive behaviour or objective truth (MRI, machine learning, etc.)—all of which might similarly show parallels to PP. (We thank the peer review for raising several of these examples.)
